# Assessment of the Needs of Caregivers of Stroke Patients at State-Owned Acute-Care Hospitals in Southern Vietnam, 2011

**DOI:** 10.5888/pcd10.130023

**Published:** 2013-08-22

**Authors:** Yumiko Hayashi, Hoang Hoa Hai, Nguyen Anh Tai

**Affiliations:** Author Affiliations: Hoang Hoa Hai, Becamex International Hospital, Thu Dau Mot City, Binh Duong Province, Vietnam; Nguyen Anh Tai, Cho Ray Hospital, Ho Chi Minh City, Vietnam.

## Abstract

**Introduction:**

Care for stroke patients has improved steadily in southern Vietnam. Medical treatments such as thrombolytic therapy have been implemented at several hospitals, and stroke-care units composed of a team of various health professionals have been created. However, little attention has been focused on providing support to caregivers of stroke patients. This study aimed to characterize the caregivers of stroke patients who were treated in state-owned acute-care hospitals and to learn about their needs when patients are discharged. Such information can be used to enhance the caregiver’s support system.

**Methods:**

We used questionnaires to conduct a descriptive study in 2011 at a state-owned acute-care hospital in southern Vietnam. We recruited study participants from among caregivers of stroke patients who had been informed of their hospital discharge date. We assessed 8 caregiver characteristics, and caregiver participants selected their needs from the survey’s list of 15 possible needs. We analyzed the data by using the independent sample *t* test and logistic regression.

**Results:**

Of the 93 caregivers who consented to participate, 86 (92.5%) completed the survey and indicated their concerns at discharge. The most frequently cited need was information on how to prevent stroke recurrence (72, 83.7%), followed by which drugs are most effective in preventing a relapse (62, 72.1%), how long recovery would take (61, 70.9%), and availability of hospitals in the patient’s hometown (60, 69.8%). A little over half of caregivers indicated financial concerns. A caregiver’s need for information on diet for a stroke survivor increased with the caregiver’s education level.

**Conclusions:**

This study revealed several needs among caregivers of stroke survivors in southern Vietnam that are similar to those found by studies of caregivers of stroke survivors in high-income countries. Our findings suggest that comprehensive stroke care that includes caregiver education about healthful diets and prevention of stroke recurrence is needed at state-owned acute-care hospitals in southern Vietnam.

## Introduction

Stroke is one of the leading causes of death in Vietnam, where noncommunicable diseases such as cancer and stroke accounted for 75% of deaths in 2008; communicable diseases accounted for only 12% of deaths (1). Historically, the prevalence of risk factors for chronic diseases has been higher in urban than in rural areas (2); however, prevalence in rural areas has been increasing (3). Urgent action is needed to address chronic diseases and their associated negative economic effects (4).

Some hospitals began implementing thrombolytic therapy for stroke patients in 2006 (5). Interprofessional stroke care units (consisting of teams of physicians, nurses, and physiotherapists) have been organized at high-ranked (by Ministry of Health of Vietnam), state-owned hospitals. However, most state-owned hospitals have patient enrollments that exceed their capacity. As a result, stroke patients are generally discharged within a week of admission, and most return to their homes in provinces where few health care resources are available. In addition, little attention is focused on supporting and educating caregivers before the patient is discharged except at one private urban hospital (FV Hospital in Ho Chi Minh City) that is supported by foreign investment. The government of Vietnam has acknowledged that shortages of health care workers affect the entire nation (1).

Other studies have found failure to provide information to caregivers of stroke patients before discharge and have also pointed to elevated levels of depression among caregivers during the acute and chronic phases of the disease (6–8). One study in a high-income country identified high levels of emotional distress among caregivers of stroke survivors and failure to meet caregiver needs (9). Our study’s objective was to assess caregiver characteristics and to evaluate caregiver education and support needs when a stroke patient is discharged from state-owned acute-care hospitals.

## Methods

### Study design

This cross-sectional study was designed to describe the sociodemographic characteristics of stroke-patient caregivers in southern Vietnam and to assess the association between these characteristics and caregiver needs when a family member (a stroke patient) is discharged from the hospital. We collected data by administering a survey at a state-owned acute-care hospital in Ho Chi Minh City from August 22 through September 1, 2011.

### Study setting and sampling

Most state-owned acute-care hospitals in southern Vietnam provide inpatient care for stroke. Few, however, focus on stroke rehabilitation during the recovery and chronic phases of the disease. Community health care services (eg, day hospitals, home care) have not yet been established at state-owned hospitals in the region. Our study was conducted at the Cho Ray Hospital in Ho Chi Minh City. We selected this hospital for the following 3 reasons: 1) it is one of the largest and highest-ranked state-owned hospitals under direct management of the Ministry of Health of Vietnam; 2) it can serve up to 2,700 inpatients, who arrive from throughout southern Vietnam, although the officially permitted number of beds is 1,800; and 3) it has been a leading teaching hospital in the region since its establishment in 1900.

### Recruitment

We planned to survey 100 caregivers. For each of 9 working days during the study period, the study team received from the hospital’s neurology department a list of stroke patients who would be discharged. A hospital social worker and the first author (Y.H.) approached all stroke patients on the list to ask if they would participate in the study. After oral and written consent were obtained, the family of each patient was asked to specify a caregiver for the patient, and the survey was administered to the specified caregiver.

### Survey instrument

We examined 8 caregiver characteristics as independent variables: place of residence (Ho Chi Minh City, southern provinces, or other), relationship to patient (core family [defined as first degree of kinship] or other), age (≤45 or ≥46); sex (male or female), education (elementary/secondary school [9 years or less of schooling], high school diploma, or higher), monthly income (≤5 million Vietnamese dong [VND] or >5 million VND), length of hospital stay, and physiotherapy for stroke survivor before discharge (yes or no) (Table 1). The 8 variables were selected on the basis of previous studies (7–21).We established the 2 age categories on the basis of Vietnamese demographics; the life expectancy in Vietnam is 73 (1). One similar (but qualitative) study in a high-income country used over or under 55 years for age of caregiver (11). One survey question “What are you worried about before discharge?” was selected as the dependent variable. Participants could choose multiple needs or concerns from a list of 15 possible needs or concerns (Table 2), most of which were derived from previous studies (7–21). The wording of the survey questions (including the translation into Vietnamese) and the appropriateness of the demographic information sought from the participants were examined and approved by the Cho Ray Hospital Ethics Committee on August 17, 2011. If a participant was not able to complete the questionnaire because of reading difficulties, the questionnaire was administered verbally. The survey was conducted in hospital space designated by the neurology department on the day of discharge or 1 day before discharge.

**Table 1:** Characteristics of Caregivers of Stroke Patients (N = 86) at State-Owned Acute-Care Hospitals in Southern Vietnam, 2011

Caregiver Characteristic	n (%)
**Place of residence**	
Ho Chi Minh City	5 (5.8)
Southern provinces	65 (75.6)
Other provinces	16 (18.6)
**Relationship to stroke patient**	
Core family^a^	68 (79.1)
Other	18 (20.9)
**Age, y**	
≤45	45 (52.3)
≥46	41 (47.7)
**Sex**	
Male	28 (32.6)
Female	58 (67.4)
**Education**	
Some elementary or secondary school	54 (62.8)
High school diploma or higher	32 (37.2)
**Income (per month), VND**	
≤5 million VND (equivalent to $250 US)	57 (66.3)
>5 million VND (equivalent to $250 US)	29 (33.7)
**Length of hospital stay^b^. days**	
2	17 (19.8)
3	14 (16.3)
4	9 (10.5)
5	20 (23.3)
6	5 (5.9)
7	6 (7.0)
8	5 (5.8)
9	1 (1.2)
10	3 (3.5)
11	1 (1.2)
12	2 (2.3)
15	1 (1.2)
20	1 (1.2)
35	1 (1.2)
**Exercise by physiotherapists before discharge and caregiver instruction in exercises**	
Yes	18 (20.9)
No	68 (79.1)

Abbreviations: VND = Vietnamese dong

a Defined as first-degree family members.

b Average length of hospital stay was 5.4 days (standard deviation, 4.5).

**Table 2:** Needs and Concerns of Caregivers of Stroke Patients (N = 86) at State-Owned Acute-Care Hospitals in Southern Vietnam, 2011

Caregivers’ Needs or Concerns	Respondents Answering Yes, n (%)
How to keep patient healthy and avoid a recurrence of stroke	72 (83.7)
Which drugs are effective to prevent recurrence and aid recovery	62 (72.1)
How long recovery will take	61 (70.9)
Which hospitals are available for health care in hometown	60 (69.8)
Patient’s ability to conduct activities of daily living at home	51 (59.3)
Decreased family income^a^	47 (54.7)
Availability of hometown physiotherapists^b^	46 (53.5)
Ability of stroke survivor to walk at home and in the community	40 (46.5)
Payment of medical fees for treatment	37 (43.0)
Caregiver’s reinstatement at work or at school	35 (40.7)
Increased burden on stroke caregivers	32 (37.2)
Recommended diet for stroke survivor	30 (34.9)
Stroke survivor’s reinstatement at work or at school	29 (33.7)
Ability of stroke survivor to communicate	26 (30.2)
Ability of stroke survivor to eat after removal of feeding tube	17 (19.8)

a Significant difference in mean for length of stay: for the group answering yes: mean, 6.28; standard deviation [SD], 5.66; for the group answering no: mean, 4.44; SD, 2.16; *t*_61_ = 2.06, *P* = .04.

b Significant difference in mean for length of stay: for the group answering yes: mean, 6.41; SD, 5.67; for the group answering no: mean, 4.33; SD, 2.18; *t*_60_ = 2.31, *P* = .02.

We asked 94 caregivers to participate in the study. Eighty-six (92.5%) indicated at least 1 need, 7 (7.5%) indicated no needs, and 1 refused to participate. Therefore, data on 8 caregivers were excluded from analysis.

### Statistical analysis

Data management and statistical analysis were performed using Predictive Analytics Software version 18 (SPSS Inc., Chicago, Illinois). First, we examined the sociodemographic characteristics of the survey participants. We then ranked each of the 15 needs or concerns according to the number of caregivers who selected it (Table 2). We used independent sample *t* tests to assess the relationship between each need or concern and mean length of hospital stay. Finally, we used logistic regression to assess the association between each need and the 8 caregiver characteristics. We calculated 95% confidence intervals (CIs).

## Results

Sixty-five (75.6%) caregivers lived in the southern provinces, and only 5 (5.8%) lived in Ho Chi Minh City (Table 1). Caregivers fell almost equally into 2 age groups: 45 (52.3%) caregivers were 45 or younger, and 41 (47.7%) were 46 or older. Fifty-four (62.8%) caregivers had some elementary- or secondary-school education (less than 9 years of schooling), and 57 (66.3%) had a monthly income of 5 million Vietnamese dong (equivalent to US $250) or less. The average length of hospital stay was 5.4 days (standard deviation [SD], 4.5). Sixty-eight (79.1%) stroke patients left the hospital without receiving any physiotherapy.

The most need most frequently expressed by caregivers was information on how to help stroke patients stay healthy and avoid a recurrence of stroke; 72 (83.7%) caregivers indicated this need (Table 2). The next most frequently identified needs were information on drugs to prevent recurrence and aid recovery (72.1% of caregivers), information on how long recovery would take (70.9% of caregivers), and availability of hospitals in hometown (69.8% of caregivers). Only a little more than half (54.7%) were concerned about decreasing family income, and only 43.0% were concerned about paying medical fees. We found a significant difference (*t*_61_ = 2.06; *P* = .04), however, in the response to concern about decreasing family income according to mean length of stay. For caregivers who did not indicate a concern about decreasing family income, the mean length of stay was 4.4 days (SD, 2.2 days). For caregivers who indicated this concern, the mean length of stay was 6.3 days (SD, 5.7 days). Forty-six (53.5%) caregivers indicated a need for physiotherapy for the stroke patient in their hometown (Table 2), whereas only 18 (20.9%) stroke patients exercised with a physiotherapist before discharge (Table 1). We found a significant difference (*t*_60_ = 2.31; *P* = .02) in response to the need for further exercise in hometowns according to mean length of stay. For caregivers who did not indicate this need, the mean length of stay was 4.3 days (SD, 2.2). For caregivers who indicated this need, the mean length of stay was 6.4 days (SD, 5.7).

None of the top 5 needs or concerns of caregivers (stroke prevention, information on drugs, recovery time, availability of hometown hospitals, and ability of stroke survivor to conduct activities of daily living) was associated with any of the caregiver characteristics (Table 3). However, place of residence, sex, education, and income were significantly associated with concern about decreasing income. The concern about payment of medical fees for treatment was significantly associated with sex (odds ratio [OR] = 3.6, 95% confidence interval [CI], 1.2–11.5) and income (OR = 0.1, 95% CI, 0.0–0.5). Length of hospital stay was an important independent determinant of the following caregiver needs or concerns: caregiver reinstatement at work or school (OR = 3.8, 95% CI, 1.1–12.8), increased burden on caregivers (OR = 3.4, 95% CI, 1.0–11.3), information on recommended foods for a healthful diet (OR = 4.5, 95% CI, 1.3–15.2), and information on stroke survivor’s ability to eat after a feeding tube is removed (OR = 6.8, 95% CI, 1.6–29.2). The more educated the caregiver, the greater the need for information on recommended foods (OR = 7.2, 95% CI, 2.2–23.8).

**Table 3:** Results of Logistic Regression Analysis (N=86)^a^_:_ Assessment of Needs of Caregivers of Stroke Patients (N=86) at State-Owned Acute-Care Hospitals in Southern Vietnam, 2011

Caregiver Needs or Concerns	Place^b^, OR (95% CI)	Sex^c^, OR (95% CI)	Education^d^, OR (95% CI)	Income^e^, OR (95% CI)	Length of stay^f^, OR (95% CI)
Prevention of recurrence	0.3 (0.1–2.0)	3.4 (0.8–14.8)	1.8 (0.4–9.6)	0.4 (0.1–1.7)	2.6 (0.5–13.5)
Drugs to prevent recurrence	1.1 (0.3–3.8)	1.2 (0.4–3.7)	0.9 (0.3–2.8)	0.7 (0.2–2.1)	2.1 (0.6–7.3)
Time to recovery	0.8 (0.2–2.9)	1.5 (0.5–4.5)	0.5 (0.2–1.6)	3.1 (0.9–10.6)	1.6 (0.5–5.6)
Availability of hospital in hometown	0.6 (0.2–2.3)	1.5 (0.5–4.6)	1.2 (0.4–4.0)	0.5 (0.2–1.5)	2.9 (0.8–10.5)
Ability to conduct activities of daily living	0.9 (0.3–3.1)	1.9 (0.6–5.5)	0.4 (0.1–1.0)	0.9 (0.3–2.6)	2.0 (0.6–6.5)
Decreased income	0.2 (0.1–0.8)	3.6 (1.1–11.1)	0.3 (0.1–0.9)	0.2 (0.1–0.7)	1.7 (0.5–6.0)
Physiotherapy	0.7 (0.2–2.1)	1.3 (0.5–3.4)	1.3 (0.5–3.6)	1.7 (0.6–4.6)	2.8 (0.9–8.4)
Ability to walk	1.9 (0.6–6.2)	2.4 (0.8–6.9)	0.5 (0.2–1.5)	0.8 (0.3–2.3)	2.8 (0.9–8.6)
Payment of hospital bills	0.5 (0.1–1.6)	3.6 (1.2–11.5)	0.6 (0.2–1.9)	0.1 (0.0–0.5)	1.7 (0.5–5.6)
Caregiver’s reinstatement at work	1.3 (0.4–4.7)	3.4 (1.1–11.1)	0.4 (0.1–1.3)	0.4 (0.1–1.2)	3.8 (1.1–12.8)
Burden on caregiver	0.4 (0.1–1.3)	1.9 (0.6–5.9)	0.6 (0.2–1.8)	0.2 (0.1–0.8)	3.4 (1.0–11.3)
Dietary recommendations	0.6 (0.2–2.1)	1.7 (0.6–5.0)	7.2 (2.2–23.8)	0.7 (0.2–2.0)	4.5 (1.3–15.2)
Stroke survivor’s reinstatement at work	0.3 (0.1–0.9)	1.9 (0.6–6.1)	0.5 (0.1–1.4)	0.3 (0.1–1.0)	1.7 (0.5–5.4)
Stroke survivor’s ability to communicate	1.4 (0.4–4.6)	1.5 (0.5–4.6)	1.2 (0.4–3.4)	0.5 (0.2–1.6)	1.4 (0.4–4.4)
Stroke survivor’s ability to eat without feeding tube	1.2 (0.3–5.5)	6.8 (1.3–36.0)	2.0 (0.6–7.4)	1.4 (0.4–5.0)	6.8 (1.6–29.2)

Abbreviations: OR, odds ratio; CI, confidence interval

a Caregiver characteristics that showed no statistical relationship to any of the needs were omitted, including relationship of caregiver to stroke patient, caregiver age, and whether patient had physiotherapy before discharge.

b Ho Chi Minh City and “other” provinces were one referent group, and southern provinces were the other.

c Referent group for sex was male.

d Referent group for education was elementary or secondary schools.

e Referent group for income was ≤5 million Vietnamese dong (equivalent to $250 US).

f Length of hospital stay was divided into 2 groups, up to five ≤5days as referent group and >6 days.

## Discussion

The study aimed to evaluate and clarify the characteristics and needs of caregivers of stroke patients at the time of discharge from state-owned acute-care hospitals. We found that caregivers in southern Vietnam were relatively young and have minimal formal education. These characteristics are nearly consistent with those observed in a previous study of northern Vietnam (3). In high-income countries, the mean caregiver age is 55 years or older (7,9,11,17). Other studies also suggest that caregiver age was an important factor to consider when tailoring support programs for them (6,11). However, we found no association between caregiver age and caregiver concerns or needs. The relatively younger age of the caregivers in our study may have influenced study results.

The most frequently expressed concerns of caregivers in our study were the same as those of caregivers in studies in high-income countries (6,8,9,13,19), which suggests a need to provide caregivers with information on preventing future strokes, local services accessible to stroke survivors, and reasonable estimates for the family of the extent to which the stroke survivor is likely to recover (8,9,19). We detected no statistical association between caregiver characteristics and the most common concerns or needs. The small sample size may not have provided sufficient power to detect such an association. However, this lack of association may suggest that, regardless of their sex, age, education, or income level, caregivers need information and services to help them with the concerns or needs assessed in our questionnaire before the stroke patient is discharged. Another study noted that the needs of many caregivers were unmet although a variety of community services were available (9). Caregiving is a multilayered activity and involves several aspects of behavior (17). Future surveys could expand the number caregiver characteristics and also consider characteristics such as age and sex of the stroke survivor.

Financial concerns or needs were expressed by only about half of the caregivers surveyed, although two-thirds of survey participants reported a monthly income at a bare livelihood level. However, the result by a univariate analysis showed that caregivers were concerned about decreased family income, particularly when the stroke patient they cared for had stayed more than 6 days at the hospital. In this study we used the characteristic “length of hospital stay” to estimate the severity of the stroke. We assumed that a hospital stay of more than 6 days indicated greater severity of the stroke and a need for further expenses for medical treatment when the stroke survivor returned home. However, multivariate analysis showed no significant association between length of hospital stay and a decrease in family income. Future surveys are expected to examine details of the relationship between length of stay and financial needs. Nevertheless, caregiver characteristics such as place of residence, sex, education, and income level should be considered when identifying those in need of financial assistance.

By using univariate analysis, we found a need for further physiotherapy following discharge when the stroke patient had been hospitalized for over 6 days; however, we found no associations by using multivariate analysis. Many issues regarding stroke rehabilitation remain unresolved in southern Vietnam. Rehabilitation departments at most state-owned acute-care hospitals, including the Cho Ray Hospital, have no inpatient beds. Few state-owned hospitals offer services for rehabilitation in recovery or chronic-care settings. Some state-owned hospitals have no rehabilitation department at all. As for rehabilitation services, educational programs in speech-language pathology were launched in Ho Chi Minh City in 2010, and physiotherapy training has been provided at some schools since 1970s. No educational programs in occupational therapy exist anywhere in Vietnam. We strongly recommend that physiotherapists at state-owned acute-care hospitals play a role in delivering relevant information on necessary physiotherapy exercises before the stroke patient is discharged. Simple distribution of pamphlets is not sufficient (6). Providing oral and printed instruction and an individualized information booklet should be considered (6,11). Conducting classes for stroke survivors and inviting caregivers to therapy sessions with the stroke survivors they care for are recommended so that caregivers can observe and learn, before discharge, which exercises the stroke survivors should do when they return home (8).

An unexpectedly noteworthy finding in the study was the association of caregiver education to need for information on recommended diets for stroke survivors. Caregivers in a previous study carried out in a high-income country had a high school or higher level of education (8). Information on lifestyle management (including creating a healthful balanced diet, reducing sodium intake, managing weight, and limiting alcohol consumption) is incorporated into stroke care as secondary prevention in high-income countries (6). Little such information is delivered before discharge from state-owned acute-care hospitals in southern Vietnam. Assessment of body mass index (BMI) has not yet been included as a routine part of health care in Vietnam. Therefore, when a stroke patient is discharged, hospital nutritionists should assume responsibility for providing caregivers with information on lifestyle management and healthful foods. Overall, our findings suggest that providing comprehensive and coordinated stroke care to stroke patients and their caregivers through teams consisting of a variety of health professionals (physicians, nurses, physiotherapists, nutritionists) is essential at state-owned acute-care hospitals in southern Vietnam.

Our study found caregiver characteristics that were consistent with those found in a study carried out in northern Vietnam (3), and the caregiver needs most frequently indicated were consistent with those found in studies conducted in high-income countries (6,8,9,13,19). Our study showed that only about half of caregiver participants expressed concern about financial needs. An unexpected finding in our study was a need for diet recommendations associated with caregiver education.

Sample bias was seen in 1 caregiver characteristic: place of residence. In addition, results were inconsistent regarding the relationship between length of hospital stay and 2 caregiver concerns: decreased family income and access to physiotherapy following discharge. These weaknesses may have led to incorrect conclusions.

Our study was conducted at only 1 state-owned acute-care hospital in southern Vietnam. Further studies are needed, particularly at other state-owned acute-care hospitals that treat residents of Ho Chi Minh City. The limited number of caregiver characteristics assessed may have influenced study results, and we found no association between caregiver characteristics and the most frequently expressed caregiver concerns. Therefore, further surveys are needed that include more caregiver characteristics (eg, physical health) and characteristics of stroke survivors (eg, age, sex, degree of walking ability).

Stroke care in southern Vietnam has improved steadily in the past decade. However, little attention has been focused on making education and support for caregivers an integral part of stroke care (an exception is one private hospital, FV Hospital, in Ho Chi Minh City that is supported by foreign investors). Our findings are consistent with those of previous studies (6,8,9,13,19): our findings suggest that state-owned acute-care hospitals in southern Vietnam should offer comprehensive, coordinated, long-term stroke care by teams of health professionals even if the hospitals are overcrowded or underfinanced. Hospital stroke care should include educating stroke patients and their caregivers in how to prevent a recurrence of stroke, providing them with information on hospitals in the patients’ hometowns, and teaching them rehabilitation exercises and the components of a healthful diet.

The incidence of stroke will increase in parallel to rapid economic growth in southern Vietnam (1). We recommend that state-owned acute-care hospitals in the region provide comprehensive and coordinated stroke care that fulfills the unmet needs of the stroke caregivers in this study. Medical professionals should be aware that caregiver education is an integral part of stroke care (6). A shift in rehabilitation philosophy from a patient-centered approach to a patient-and-caregiver approach may be necessary (17). The following interventions described in previous studies (6–8,11,13,21) may be helpful in structuring feasible stroke care for patients at discharge: 1) maximize the role of social workers as financial consultants and the role of nutritionists as diet consultants 2), provide a contact person that caregivers can call anytime for advice 3), provide written materials on the most common concerns or needs 4), conduct classes for both stroke survivors and their caregivers in how to self-manage (eg, how to prevent falls, use the toilet, exercise at home) 5), invite stroke survivors and caregivers to physiotherapy sessions to help them learn by observing other survivors and caregivers. However, zero-sum competition for scarce health care resources (one patient’s win is another’s loss) must be avoided (22,23). Creating and improving caregiver support for some people should not be accomplished by taking resources away from other people.
